# Kangxianling formula attenuates renal fibrosis by regulating gut microbiota

**DOI:** 10.1186/s40001-024-01778-8

**Published:** 2024-03-18

**Authors:** Pengyu Tao, Haiyan Liu, Guangjian Hou, Jianrao Lu, Yukun Xu

**Affiliations:** 1 Department of Nephrology, Seventh People’s Hospital Affiliated to Shanghai University of Traditional Chinese Medicine, Shanghai, China; 2 Department of Ultrasound, The Affiliated Taian City Central Hospital of Qingdao University, Taian, China; 3 Shandong University of Traditional Chinese Medicine, Jinan, China; 4 Department of Geriatric Medicine, Affiliated Hospital of Shandong University of Traditional Chinese Medicine, Jinan, China

**Keywords:** Kangxianling formula, Renal fibrosis, UUO rat, Gut

## Abstract

**Background:**

Renal fibrosis (RF) produced adverse effect on kidney function. Recently, intestinal dysbiosis is a key regulator that promotes the formation of renal fibrosis. This study will focus on exploring the protective mechanism of Kangxianling Formula (KXL) on renal fibrosis from the perspective of intestinal flora.

**Methods:**

Unilateral Ureteral Obstruction (UUO) was used to construct rats’ model with RF, and receive KXL formula intervention for 1 week. The renal function indicators were measured. Hematoxylin–eosin (HE), Masson and Sirus red staining were employed to detect the pathological changes of renal tissue in each group. The expression of α-SMA, Col-III, TGF-β, FN, ZO-1, and Occuludin was detected by immunofluorescence and immunohistochemistry. Rat feces samples were collected and analyzed for species’ diversity using high-throughput sequencing 16S rRNA.

**Results:**

Rats in UUO groups displayed poor renal function as well as severe RF. The pro-fibrotic protein expression in renal tissues including α-SMA, Col-III, TGF-β and FN was increased in UUO rats, while ZO-1 and Occuludin -1 expression was downregulated in colon tissues. The above changes were attenuated by KXL treatment. 16S rRNA sequencing results revealed that compared with the sham group, the increased abundance of pathogenic bacteria including *Acinetobacter*,* Enterobacter* and *Proteobacteria* and the decreased abundance of beneficial bacteria including *Actinobacteriota, Bifidobacteriales, Prevotellaceae, and Lactobacillus* were found in UUO group*.* After the administration of KXL, the growth of potential pathogenic bacteria was reduced and the abundance of beneficial bacteria was enhanced.

**Conclusion:**

KXL displays a therapeutical potential in protecting renal function and inhibiting RF, and its mechanism of action may be associated with regulating intestinal microbiota.

## Introduction

Renal fibrosis (RF), a vital pathological factor, produces a negative influence on renal function that accelerates the progress of chronic kidney diseases to renal failure [1]. The over accumulation of extracellular matrix in renal tissues, resulting from various factors, promotes the development of renal fibrosis [2]. The degree of renal interstitial fibrosis is the most important factor affecting renal function. Therefore, renal fibrosis has received increasing attention from researchers. Exploring effective measures to attenuated renal fibrosis is crucial in improving overall health of patients with kidney diseases [3]. Traditional Chinese medicine (TCM) has a long history in the treatment of renal fibrosis, and their ant-renal fibrosis effect is evidenced by inhibiting renal fibrosis through multi-targets and multi-pathways [4, 5].

Intestinal flora refers to the microbial communities that maintain a relative balance, interdependence, and mutual restriction of various qualities and quantities resident in the gastrointestinal tract of the human digestive system [6]. The homeostasis of gut microenvironment plays a crucial role in human health [7]. Recently, amounting evidence suggested that intestinal flora dysbiosis is a key contributor to renal fibrosis [8]. Kidney disease can induce the alterations in intestinal flora, which contributed to the aggravation of RF [9, 10]. Studies have shown that intestine pathogenic bacteria including *Acinetobacter*,* Enterobacter* and *Proteobacteria* are key target bacteria for renal fibrosis, and their biological functions are related to inflammation, immunity, and kidney function [11–13]. The mechanism of TCM on regulating intestinal diseases is related to its effective components regulating the imbalance of intestinal flora through direct or indirect effects [14]. These components are absorbed and exert their effects after metabolism by intestinal flora, which helps restore the balance of intestinal microecological system [15].

The preliminary research found that the Kangxianling formula (KXL) can alleviate the fibrosis process by inhibiting TGF-β/Smads pathway [16]. Currently, there are few reports on the study of mechanism of KXL regulating intestinal flora. Based on the previous research, this experiment will focus in the roles of KXL in regulating intestinal flora, providing more comprehensive experimental evidence for revealing the anti-RF mechanism of KXL.

## Materials and methods

### KXL decoction preparation and main reagents

KXL granules contain five drugs: *Radix achyranthis bidentatae* (Niuxi 15 g), *Rheum officinale* (Dahuang 15 g), *Salvia miltiorrhiza* (Danshen 15 g), *Peach kernel* (Taoren 15 g) and *Astragalus membranaceus* (Huangqi 15 g), which were purchased from Shanghai seventh people’s hospital. All these granules were dissolved in boiled water.

BCA Protein Assay Kit (Beyotime, China), and PVDF membrane, protein marker, and SDS-PAGE were purchased from Servicebio Company (China). Primary antibodies and secondary antibody including Fibronection (FN, GB114491), Transforming growth factor beta 1 (TGF-β1, GB11179), Occludin (GB111401), ZO-1 (GB111402), Collagen-III (Col-III, GB111629) and α-smooth muscle actin (α-SMA, GB12045) were purchased from Servicebio Company (China).

### Animal model

24 SPF grade SD rats (120–150 g) were acquired from Hangzhou Ziyuan Experimental Animal Technology Co. Ltd. (Hangzhou, China) and housed in animal center of Shanghai Seventh People’s Hospital with free access to food and water. All rats were randomly divided into 4 groups: Sham group (*n* = 6), UUO group (*n* = 6), KXLM (middle dose: 15.6 g/kg) group (*n* = 6) and KXLH (high dose: 31.2 g/kg) group (*n* = 6). Unilateral Ureteral Obstruction model (UUO) was employed to mimic the progress of RF. The UUO protocol was described briefly as follows. The 4% sodium pentobarbital (1.5 ml/kg, Shanghai Pharmaceutical Factory, Shanghai, China) was used to anesthetize rats by intraperitoneal injection, the left ureter was separated and ligated at the renal hilum and distal end of the ureter with 4–0 silk. Rats in the sham group underwent an identical procedure to the UUO rats, with the exception of not ligating the ureter. All rats were killed at 7th day after 6 consecutive days. The renal tissues and blood were collected for further biological detection. The animal study was complied with the items of Animal Ethics Committee of Shanghai Seventh People’s Hospital (PZSHUTCM220711030).

### Biochemical indicator detection

The blood samples were collected from the abdominal aorta and related biochemical indicators were tested, including blood urea nitrogen (BUN), serum creatinine (SCr) by automatic biochemical analyzer. The urine samples were collected from the metabolic cages before the rats were killed, and 24 h urine protein were measured by urine protein kit.

### Pathological detection

The renal tissues and colon tissues were fixed in a 4% paraformaldehyde solution, dehydrated with gradient alcohol, made transparent with xylene, and embedded in paraffin. The samples were sliced with thickness of 5 µm and stained with hematoxylin eosin (H&E), Masson’s trichrome (Masson) and Sirius red staining. The morphology of renal tissues and colon tissues was observed under microscope.

### Immunohistochemistry (IHC)

The procedure of Immunohistochemistry was described in our published paper [16]. The renal tissues were incubated with primary antibodies (FN and TGF-β1), the colon tissues were incubated with Occludin. The positive area of FN, TGF-β1 and Occludin were measured by image J.

### Immunofluorescence analysis (IF)

After the procedure of dewaxing, hydration, endogenous peroxidase inactivation and blocking with sheep serum, the renal tissues were incubated with primary antibodies Col-III and α-SMA. The colon tissues were incubated with ZO-1 and E-cadherin. The renal tissues were then stained with DAPI (G1012, Servicebio, China) and observed under confocal microscope.

### 16S rDNA amplicon sequencing analysis process

The raw sequencing data in FASTQ format were preprocessed using cutadapt to remove adapters from paired-end reads. After trimming, low-quality sequences were filtered out, and the reads were denoised, merged, and chimeras were detected and excised with DADA2 and QIIME2's default parameters. Indices such as simpson, Chao1, ACE, and shannon were employed to perform α-diversity analysis. For β-diversity analysis, Principal Coordinate Analysis (PCoA) was conducted. In addition, species composition analysis was performed at the phylum order and family levels.

### Statistical analysis

All experimental data are presented as the mean ± standard error. GraphPad Prism 6 software (GraphPad, CA, USA) was used for statistical analysis and image construction. *P* < 0.05 was considered statistically significant.

## Results

### KXL improved renal function and attenuated renal injuries

**Fig. 1:**
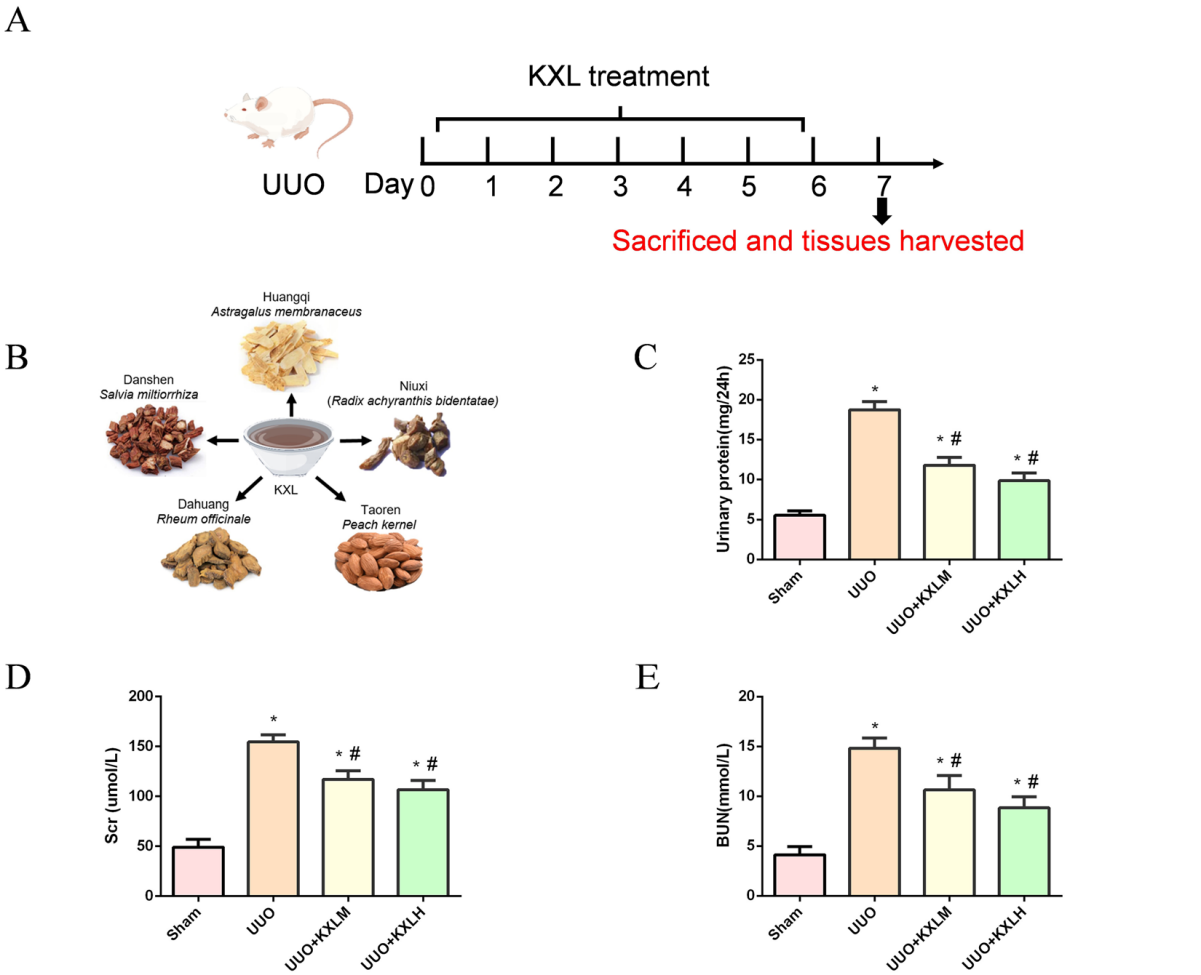
**A** The treatment duration for UUO rats. **B** The composition of KXL. **C**–**E** The renal function of all groups. *P < 0.05 *vs* sham group; # < 0.05 vs UUO group

**Fig. 2:**
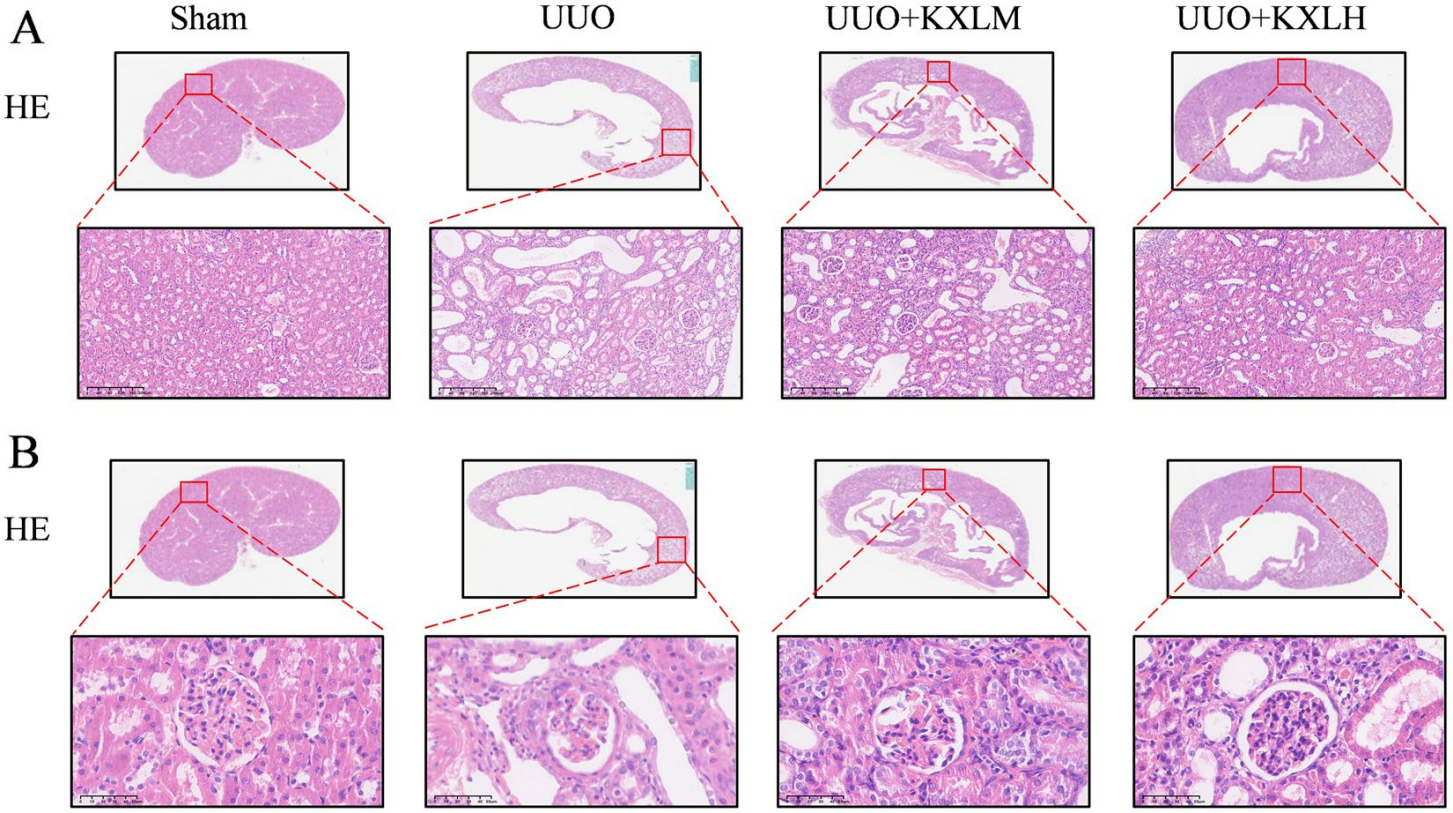
**A** HE staining of glomeruli of rats in all groups, scale bar 100 µm. **B** HE staining of renal tubule of rats in all groups, scale bar 100 µm

The composition of KXL and the treatment duration is shown in Fig. 1A, B. The levels of BUN, Scr, and 24-UP levels were increased in the UUO group compared with the Sham group, but were reduced in both KXL medium and high dose groups (Fig. 1C–E). HE (Fig. 2A, B) staining showed that there were no abnormalities in the glomerulus, tubule, and interstitial of the sham operation group. In the UUO group, the renal tubules were dilated or contracted, and ECM were accumulated in the glomerulus, accompanied by partial glomerular atrophy or compensatory hypertrophy; tubular epithelial cells swelled and shed, and some lumens showed obvious dilation, accompanied by infiltration of inflammatory cells. The above pathological injuries were alleviated by being treated with KXL formula, indicating that KXL formula possessed the effect of protecting renal function and alleviating renal injury.

### KXL attenuated the formation of renal fibrosis

**Fig. 3:**
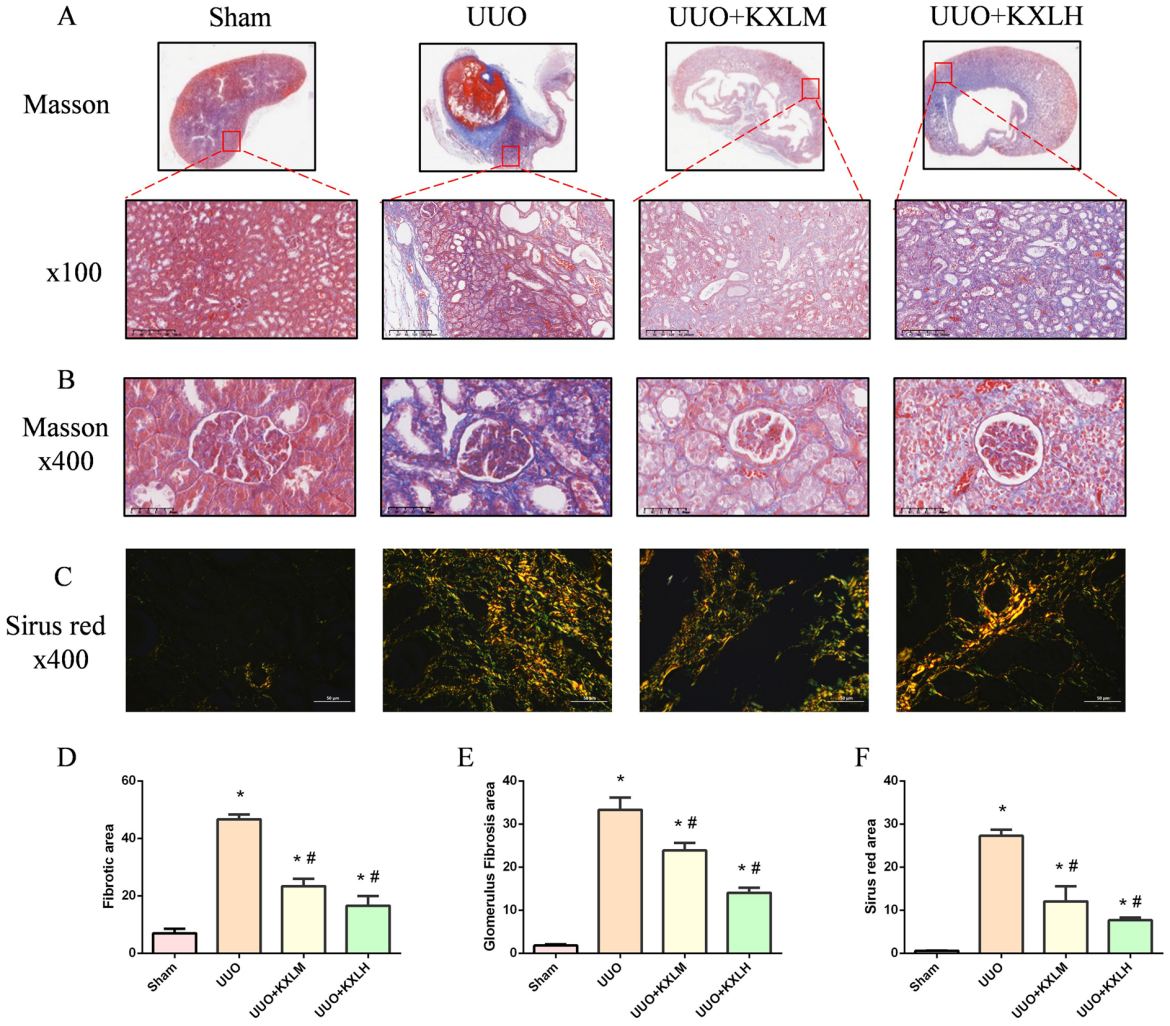
**A** Masson staining of renal tissues. **B** Masson staining of glomerulus. **C** Sirus red staining of renal tissues. **D**–**F** Positive area of fibrotic area. *P < 0.05 vs sham group; # < 0.05 *vs* UUO group

Both Masson and Sirus red staining (Fig. 3A–C) revealed that compared with the Sham group, abnormal collagen fiber proliferation was observed in the renal tubule interstitial area from the UUO group, and large areas of blue collagen fiber deposition were observed, indicating the area of renal tissue fibrosis in the UUO rats were significantly expanded (*P* < 0.05) (Fig. 3D–F). But the area of renal fibrosis in UUO rats treated with KXL formula gradually decreased (*P* < 0.05) (Fig. 3D–F), indicating that KXL formula produced the effect of delaying the disease progression of RF and protecting renal tissue.

### KXL treatment inhibited the expressions of RF-related proteins

**Fig. 4:**
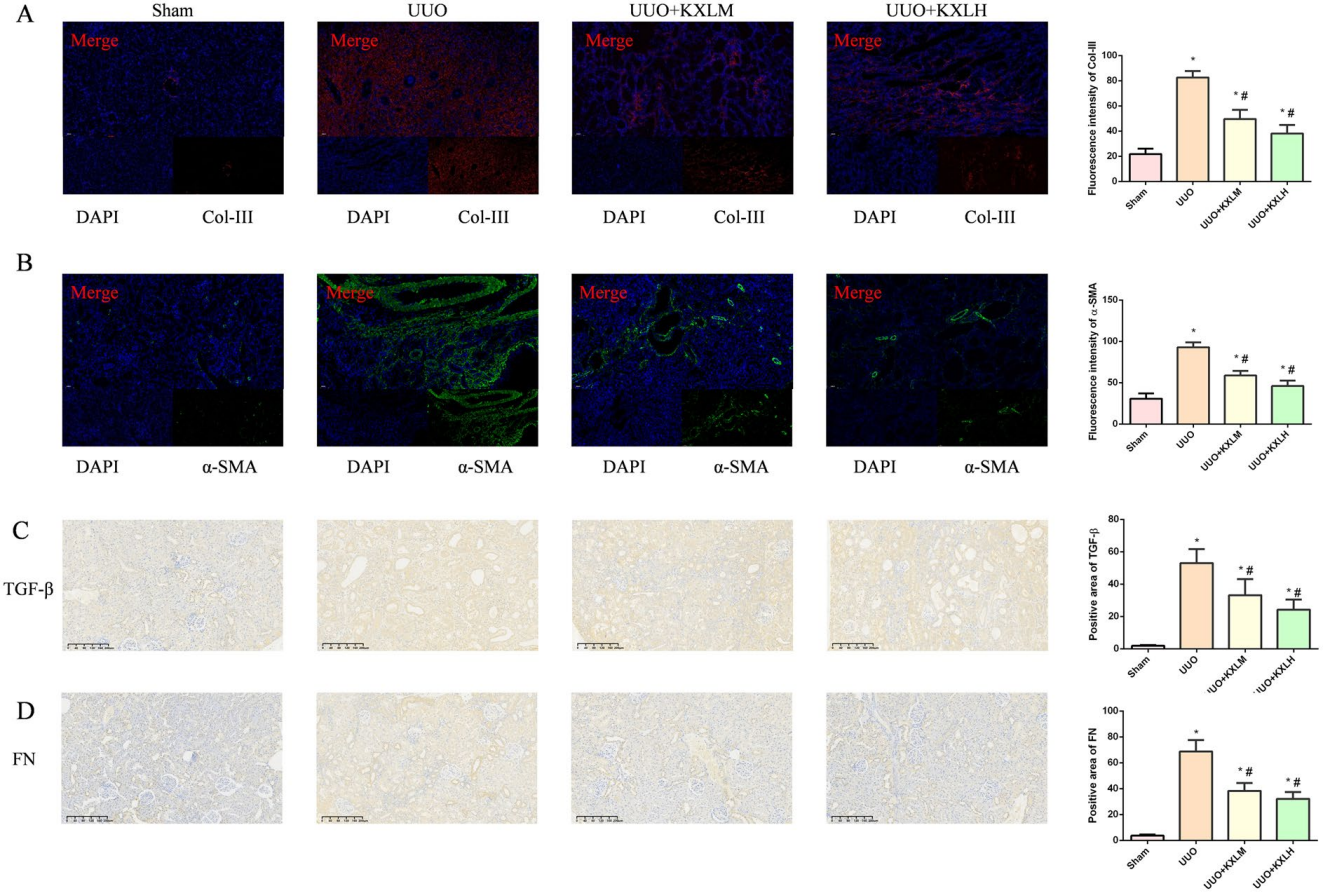
**A**, **B** The immunofluorescence analysis of α-SMA and COL-III expression in renal tissues. **C**, **D** The immunohistochemistry analysis of FN and TGF-β expression in renal tissues. *P < 0.05 vs sham group; # < 0.05 *vs* UUO group

α-SMA, TGF-β and FN are important markers of RF. The activation of α-SMA, TGF-β, and FN were contributed to promote the synthesis of collagen, accelerating the process of fibrosis. Col-III is the main component of ECM, and its expression level is closely related to the degree of organ fibrosis. IHC results (Fig. 4C, D) indicated that compared to the Sham group, the renal tissues of rats subjected to UUO exhibited a sharp increase in the positive area of TGF-β and FN, which could be reduced by KXL treatment. IF (Fig. 4A, B) results showed that compared to the Sham group, the renal tissues of rats subjected to UUO exhibited a sharp increase in the fluorescence intensity of α-SMA and Col-III, which could be reduced by KXL treatment.

The above results indicate that KXL exerted anti-fibrotic effect by suppressing activation of α-SMA, TGF, FN and Col-III.

### KXL alleviated intestinal mucosal damage

**Fig. 5:**
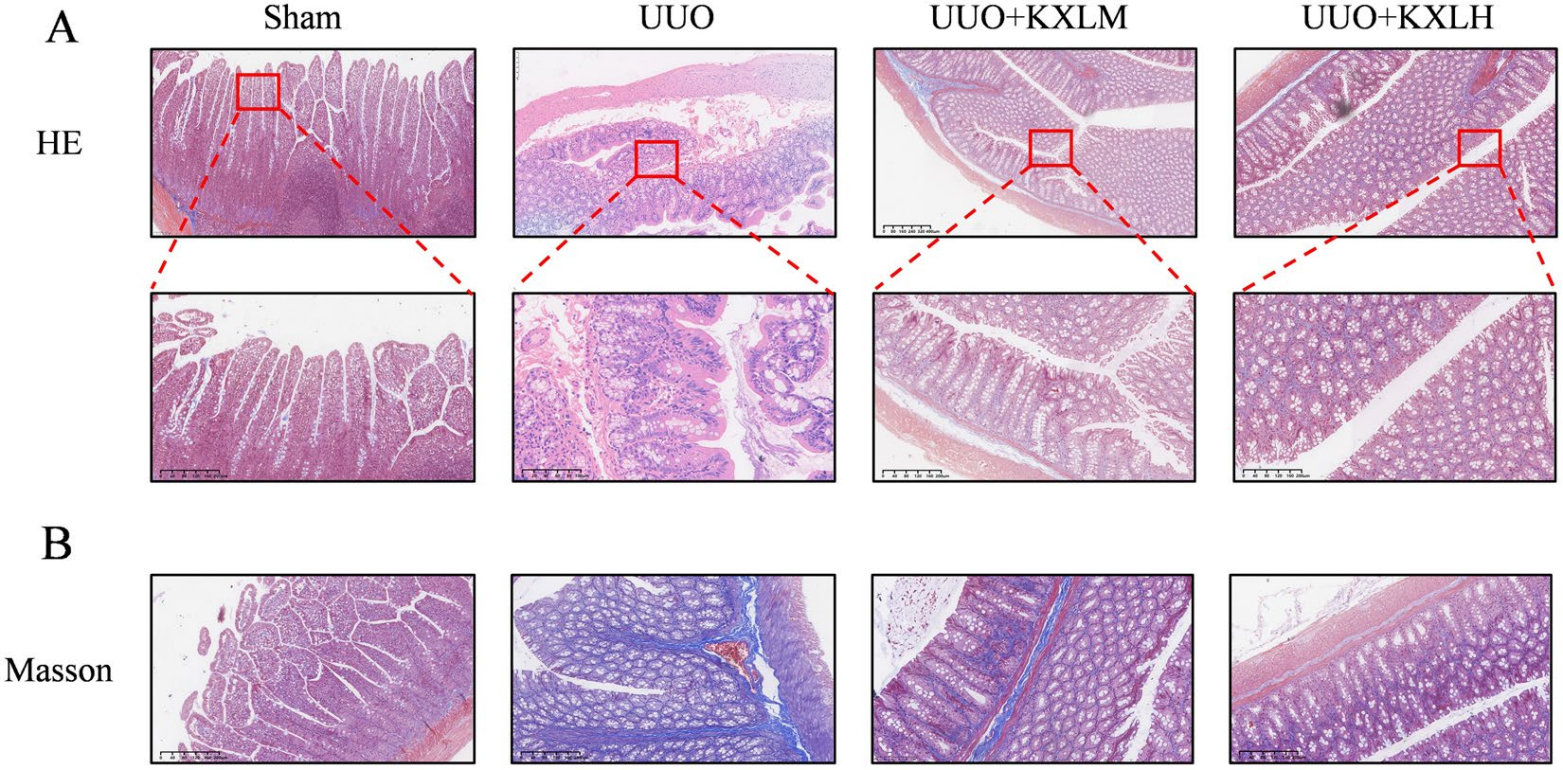
**A** HE staining of colon tissues of rats in all groups. **B** Masson staining of colon tissues of rats in all groups

Figure 5A and B reflects that in the Sham group, the structure of the serous layer, muscular layer, submucosal layer, and mucosal layer of the colon tissue was clear, the glandular body was arranged neatly, containing a large number of goblet cells, and the mucosal surface was covered with a large number of villi; However, in the UUO group, we found that the structure of colon was disordered, the glandular body was obviously damaged, and massive amount of inflammatory cells’ infiltration. But after KXL treatment, the intestinal mucosal epithelium was intact, the glandular body structure was arranged neatly, and less inflammatory cells’ infiltration was observed.

### KXL maintained the integrity of intestinal barrier

**Fig. 6:**
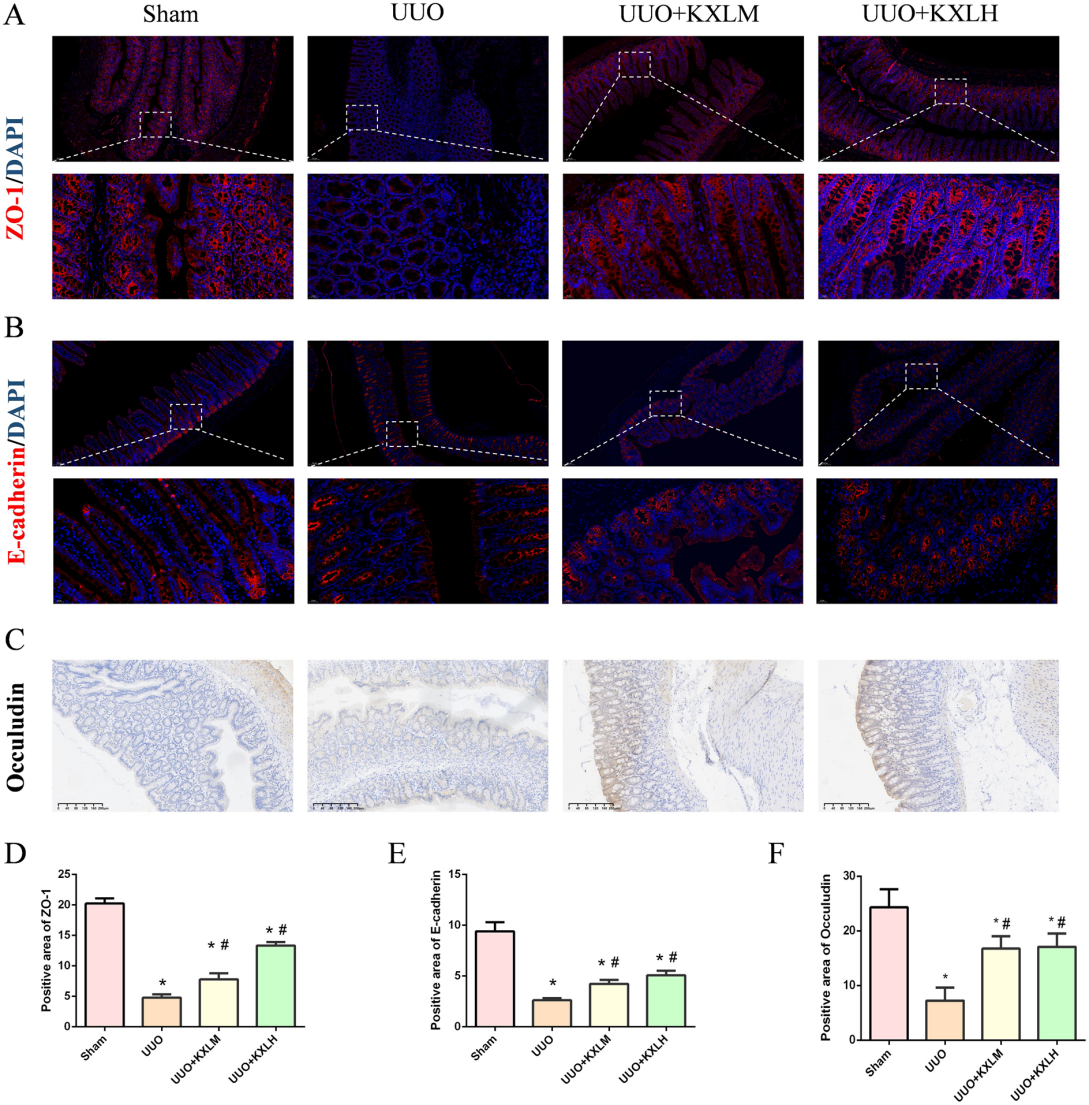
**A** IF detected the expression of ZO-1. **B** IF detected the expression of E-cadherin. **C** IHC detected the expression of Occuludin. **D**–**F** The positive area of ZO-1, E-cadherin and Occuludin calculation. *P < 0.05 vs sham group; #< 0.05 vs UUO group

E-cadherin, Occuludin and ZO-1 are three vital intestinal tight junction proteins essential for protecting integrity of intestinal barrier. The reduced expressions of Occuludin and ZO-1 are contributed to the loss of integrity of the intestinal barrier. IHC results (Fig. 6C, F) showed that colon tissues of UUO rats suffered a decreased positive area of Occuludin compared with the rats in Sham group, which could be restored by KXL treatment. IF results showed the colon tissues of UUO rats displayed less fluorescence intensity of ZO-1 (Fig. 6A, D) and E-cadherin (Fig. 6B, E) compared with the sham group, which could be upregulated by KXL intervention. These outcomes indicate that KXL can repair the damaged intestinal barrier permeability by restoring the expressions of intestinal tight junction proteins.

### The regulatory effect of KXL on gut microbiota diversity

**Fig. 7:**
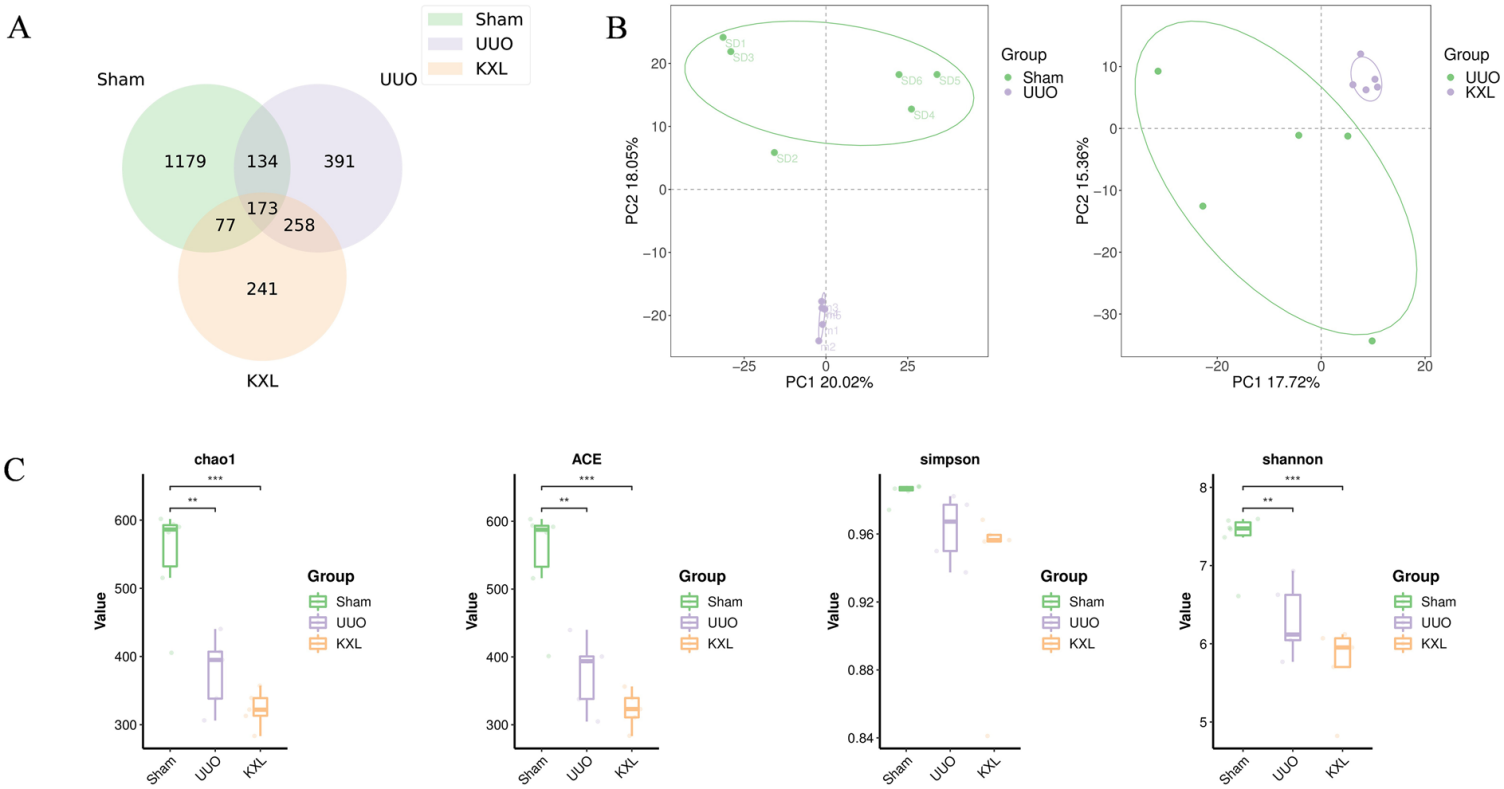
**A** Venn diagram of Sham group, UUO group and KXLH group. **B** The PCA of Sham group, UUO group and KXLH group. **C** The Chao, Ace, Simpson and Shannon of Sham group, UUO group and KXLH group

The Venn diagram (Fig. 7A) displayed that there were 2453 OUTs across all three groups, including 1563 OUTs in the Sham group, 956 OUTs in the UUO group, and 749 OUTs in the KXLH group. Notably, 173 of these OUTs were shared among all three groups. Subsequently, we used α diversity analysis to analyze the diversity of gut microorganisms. As shown in Fig. 7B, C, compared to Sham group, the Chao, Ace, Simpson and Shannon indices in the UUO rats were significantly reduced. However, after KXL intervention, the aforementioned indices in the KXL group displayed a further decrease.

**Fig. 8:**
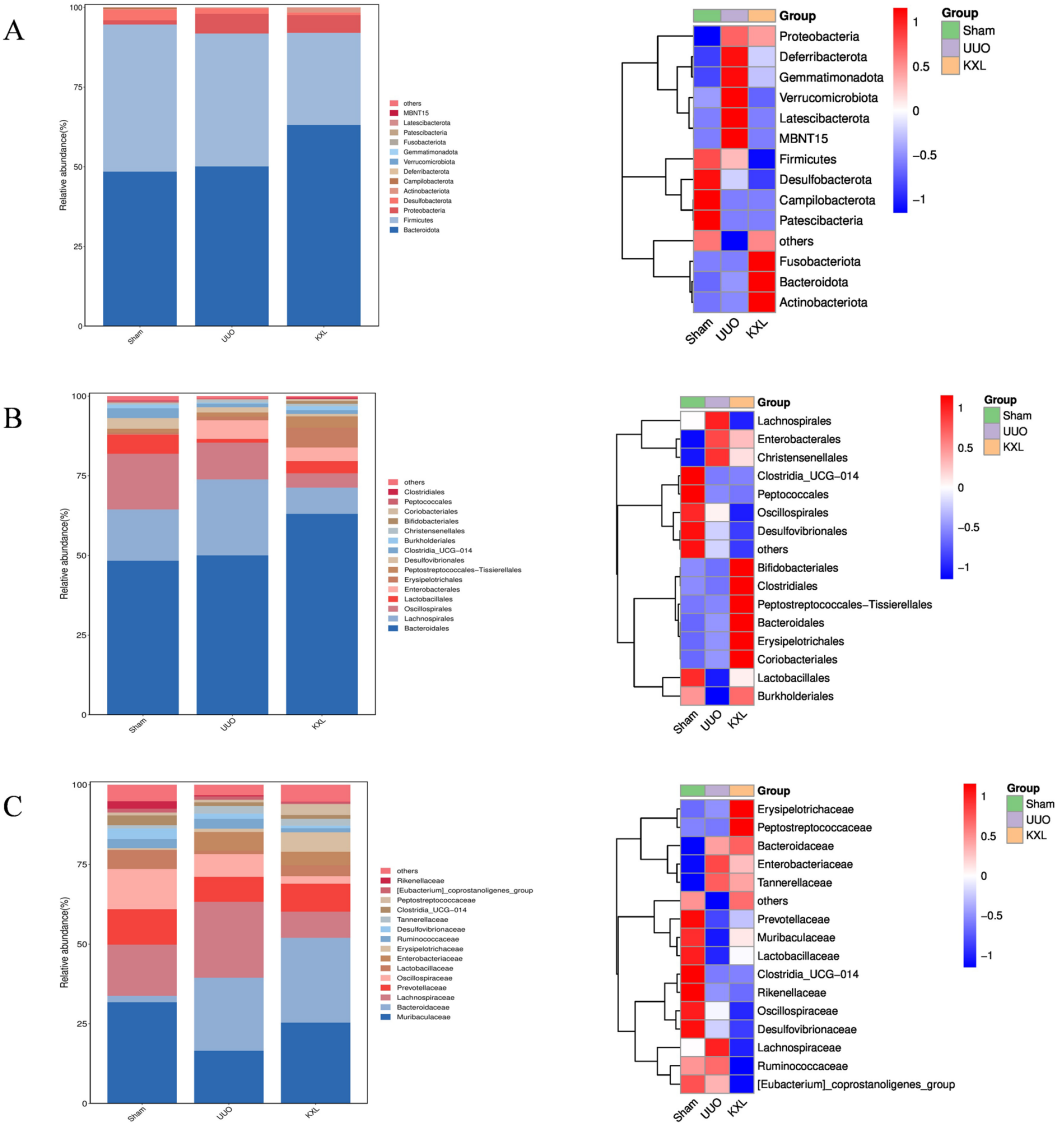
The analysis of gut microbiota composition revealed that at the phylum **A**, order **B** and family level **C**, respectively

The in-depth analysis of gut microbiota composition revealed that at the phylum level (Fig. 8A), *Bacteroidota, Firmicutes, Proteobacteria* and *Desulfobacterota* were identified the most occupied gut microbiota in Sham, UUO and KXLH group. In comparison with the Sham group, a higher abundance of *Proteobacteria* and a lower abundance of *Actinobacteriota* were found in rats subjected to UUO. After KXL intervention, the abundance of *Proteobacteria* was reduced, while the abundance of *Actinobacteriota* was increased. At order level (Fig. 8B), *Lachnospirales, Oscillospirales* and *Lactobacillales* were identified the most occupied gut microbiota in Sham, UUO and KXLH group. In comparison with the Sham group, a lower abundance of *Lachnospirales* and *Lactobacillales* was observed in UUO rats, which could be increased by KXL intervention. At family level (Fig. 8C), in comparison with the Sham group, a lower abundance of *Prevotellaceae* and a higher abundance of *Ruminococcaceae* was observed in UUO rats. But KXL intervention could reduce abundance of *Ruminococcaceae* and increase abundance of *Prevotellaceae*.

### LEfSe analysis

**Fig. 9:**
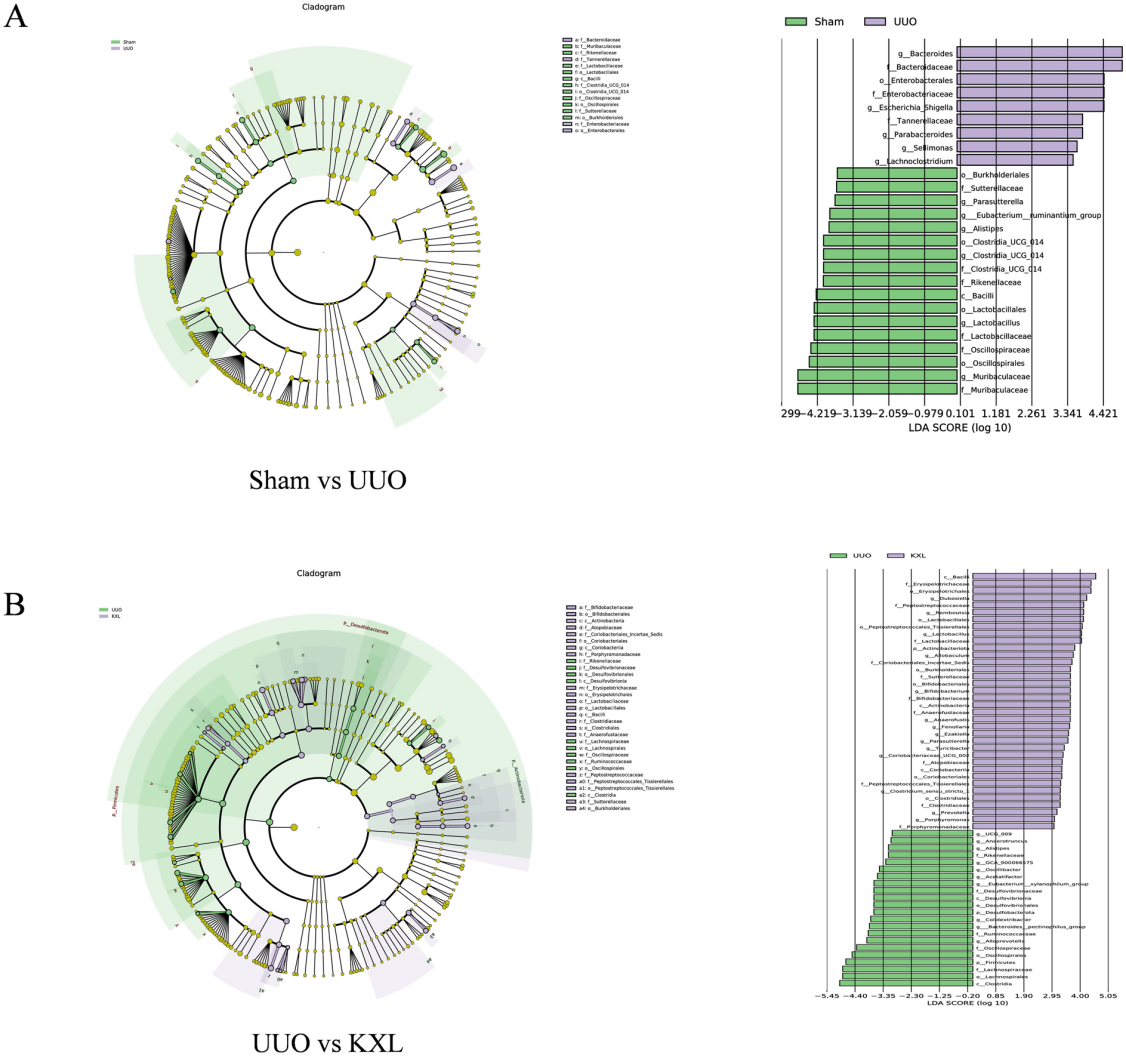
**A** The LEfSe analysis of Sham group and UUO group. **B** The LEfSe analysis of UUO group and KXLH group

LEfSe analysis can select species or genes that exhibit significant differences and elucidate their distinctions, making it a crucial application in the analysis of gut flora. By utilizing LEfSe analysis, it becomes possible to pinpoint biomarker species that are linked to disease states, providing deeper insights into the function of gut flora in the different stages of diseases development. As shown in Fig. 9A, B, the main bacteria communities of UUO group were *Enterobacteriaceae, Ruminococcaceae, Desulfobacterota, Acetatifactor, Rikenellaceae* and *Alistipes*, which are pathogenic bacteria that promotes the release of pro-inflammatory factors contributing to the disruption of gut barrier. While *Actinobacteria, Lactobacillales, Burkholderiales, Bifidobacteriales* and *Prevotella*, the important beneficial bacteria were identified in the KXL group that promote the production of SCFAs conferring health benefits on host.

## Discussion

Targeting kidney fibrosis treatment still remains a significant clinical challenge [17]. Current medicine lacks a specific treatment for RF. Inhibition of RF progression has a positive impact in maintaining normal kidney function at earlier period. Pathological experimental results demonstrate that KXL treatment can effectively mitigate renal fibrosis and improve renal function in in rats subjected to UUO. α-SMA, TGF-β, FN and Col-III are vital renal fibrosis-related proteins [18]. The activation of α-SMA, TGF-β, FN and Col-III caused by various factors contributed to exacerbation of organ fibrosis [19]. Col-III, a major component of ECM, and high expression of Col-III foster the generation of collagen fibers leading to ECM deposition in the kidney tissue and expediting renal fibrosis progression [20]. As shown in IHC and IF assays, it is evident that KXL treatment could block the production of α-SMA, TGF-β, FN and Col-III. These results further confirm the objectivity of this study on KXL’s treatment against RF.

The pathogenesis of renal fibrosis is intricate, encompassing the activation of numerous proteins [21]. α-SMA, a crucial cytoskeletal protein in the renal fibrosis process, is mainly expressed in renal interstitial fibroblasts [22]. Harmful factors like oxidative stress and inflammatory responses can influence the expression level of α-SMA, fueling the transformation of fibroblasts into myofibroblasts [23]. Overexpression of α-SMA can trigger fibroblast proliferation and the synthesis of collagen protein Col-III. As the primary component of the extracellular matrix, collagen is excessively deposited in renal fibrosis, causing renal structure destruction and functional loss, ultimately speeding up the renal fibrosis process [24]. TGF-β is a multifunctional cytokine that holds a pivotal position in renal fibrosis. TGF-β can stimulate fibroblast proliferation and collagen synthesis, thus promoting the progression of renal fibrosis [25]. FN (Fibronectin) is an extracellular matrix protein, which also plays a significant role in renal fibrosis [26]. FN interacts with TGF-β to form a complex that fosters collagen synthesis and extracellular matrix accumulation, ultimately fueling the renal fibrosis process [26].

The intestinal mucosa acting as a main site for communication between the internal and external environments performs multiple functions, including mechanical, immune, biological and chemical barrier [27]. It serves as the major defense system in maintaining the balance of the gut microbiota. Under a normal physiological state, the intestinal lumen contains a significant number of bacteria and endotoxins; however, their blood concentration remains low and they do not cause any disease symptoms [28]. This is attributed to the intact intestine barrier function regulated by ZO-1 and Occludin, which effectively restricts the entry of macromolecules like bacteria, toxins, and inflammatory factors into the bloodstream [29, 30]. However, if intestine barrier is disrupted, the decline in the expressions of ZO-1 and Occludin leads to the enhanced intestinal mucosa permeability. This allows leakage of bacteria and toxins from the intestine into the bloodstream, resulting in LPS entering the blood circulation, which result in the development of chronic metabolic endotoxemia in the body [31]. Furthermore, a disruption in the immune barrier can lead to a decrease in the secretion of SIgA by intestinal wall lymphocytes. This decrease in SIgA secretion diminishes the anti-inflammatory ability and promotes pathogenic bacteria translocation that leads to host inflammation [32]. Therefore, maintaining the integrity of both mechanical and immune barriers is crucial to maintain gut health.

Multiple studies have confirmed that disorders in the intestinal flora are a significant factor in kidney disease [33]. It has been discovered that the colonized bacteria in the human intestine can be categorized into nine phyla that are the dominant bacterial groups, accounting for up to 90% of the total [14]. Under normal circumstances, the mutualistic symbiotic relationship between the intestinal flora, the human body, and the external environment maintains a dynamic balance. On one hand, gut microbiota participates in the process of nutrients intake such as vitamins in the body. On the other hand, gut microbiota can regulate immune function, defend against pathogenic bacteria invading the intestinal mucosa and maintain the relative stability of the internal environment [34].

Patients with kidney disease exhibited alterations in the composition of their intestinal microbiota, along with corresponding alterations in gut metabolites. These gut metabolites usually exist in the form of uremic toxin compounds. Accumulating pieces of evidence have reported that the abundance of *Acinetobacter, Enterobacter, and Proteobacteria* is significantly increased in the feces of CKD patients than those of healthy individuals, while the abundance of *Actinobacteriota* decreases [35]. These pathogenic bacteria can facilitate the decomposition of nitrogen-containing organic substances in the intestine, resulting in the production of various potential toxins such as ammonia, amines, phenols and indoles. These toxic substances damage the intestinal barrier and further exacerbate kidney injury being absorbed into the bloodstream [36]. Our study found that abundance of pathogenic bacteria including *Proteobacteria* and *Enterobacter* were increased in the UUO group, but the abundance of Actinobacteriota decreased. However, after KXL intervention, the abundance of pathogenic bacteria can be reduced while increasing the abundance of *Actinobacteriota*.

Recent studies have reported that SCFAs (short-chain fatty acids) confer numerous health benefits to host. *Lactobacillales, Ruminococcaceae, Lachnospirales* and *Prevotellaceae* are the main SCFAs producing bacteria [37]. SCFAs play a pivotal role in regulating intestinal health by modulating chemical, physical and immune barriers. SCFAs such as butyrate can repair and enhance the intestinal barrier function [38]. In addition, butyrate also regulates the expression of the tight junction protein to safeguard the integrity of intestinal barrier and consequently reducing intestinal permeability. Our study found that abundance of producing bacteria SCFAs *Lactobacillales, Ruminococcaceae, Lachnospirales and Prevotellaceae* were decreased in the UUO group, which could be rescued by KXL intervention.

The study still has some limitations need to be addressed. First, due to the complexity of herb medicine, the specific composition of KXL should be identified. Second, a germ-free or germ knock out animal model should be employed to validate the positive influence of KXL on restoring the balance of gut microenvironment. Last, the study is only based on animal experimental research and cannot reflect the actual situation in human body; human samples will better reflect the efficacy of KXL. Addressing the limitations mentioned above will do us a great favor in elucidating the anti-fibrotic effect of KXL.

## Conclusion

Our study revealed that renal protective mechanism KXL may be associated with blocking pro-fibrotic proteins expression, while repairing intestinal mucosal barrier to reduce intestinal permeability, and simultaneously reducing the proliferation of pathogenic bacteria and upregulating beneficial flora, especially the pro-SCFAs producing flora, thereby inhibiting the release of inflammatory factors.

## Data Availability

The datasets are available from the corresponding author on reasonable request.
